# American System of Dentistry

**Published:** 1887-03

**Authors:** 


### AMERICAN SYSTEM OF DENTISTRY.

We have received from the publishers, Lea Brothers & Co., the second volume of this work, which includes operative and prosthetic dentistry. The papers which it contains are by Henry A. Baker, Alonzo P. Beale, A. G. Bennett, C. F. W. Bödecker, W. G. A. Bonwill, Theodore F. Chupein, W. W. Evans, S. H. Guilford, William R. Hall, A. W. Harlan, Robert S. Ivy, Louis Jack, Edward C. Kirk, Wilbur F. Litch, S. B. Palmer, D. D. Smith, Thomas. C. Stellwagen, William H. Trueman, James Truman and George W. Weld. When the whole is in our hands we shall give to it the attention and space which its importance demands, but it is impossible to review so comprehensive a work by piecemeal. We can, however, say that it is a very handsome volume indeed.

